# Gender Differences in Gut Microbiome Composition Between Schizophrenia Patients With Normal Body Weight and Central Obesity

**DOI:** 10.3389/fpsyt.2022.836896

**Published:** 2022-03-15

**Authors:** Yun-Lin Tsai, Yen-Wenn Liu, Peng-Nien Wang, Chun-Yuan Lin, Tsuo-Hung Lan

**Affiliations:** ^1^Tsaotun Psychiatric Center, Ministry of Health and Welfare, Nantou, Taiwan; ^2^Institute of Biochemistry and Molecular Biology, National Yang Ming Chiao Tung University, Taipei, Taiwan; ^3^National Changhua University of Education, Changhua, Taiwan

**Keywords:** schizophrenia, microbiome, central obesity, gender difference, gut dysbiosis

## Abstract

**Background:**

Obesity is a common health problem among patients with schizophrenia, but the precise mechanisms are not fully understood. There has been much interest in the relationship between gut microbiome and development of obesity. Gender-dependent microbial alteration has been reported in previous studies. However, the gender factor in gut microbiome composition of schizophrenia patients has been less investigated. Our study aimed to identify differences in gut microbiota between schizophrenia patients with normal weight and central obesity and investigate the gender specific features.

**Method:**

Twenty participants (10 males, 10 females) with central obesity (CO) and 20 participants (10 males, 10 females) with normal weight (NW) were recruited from two rehabilitation wards in a psychiatric hospital in central Taiwan. Fecal samples from 40 participants were processed for microbiota analysis. The intestinal microbiota composition was analyzed using next-generation sequencing and QIIME software.

**Results:**

Significantly higher richness of gut microbiota at the class level (measured by the number of observed OTUs) was observed in female NW subjects than in female CO subjects (*P* = 0.033). Furthermore, female NW subjects showed higher alpha diversity at both phylum and class levels (measured by the Shannon, Simpson, and Inverse-Simpson indexes) compared with female CO subjects. Males showed no significant difference in alpha diversity between groups. Taxonomic analysis showed that female CO subjects had significantly lower abundance of Verrucomicrobia (*P* = 0.004) at the phylum level, reduced abundance of *Akkermansia* (*P* = 0.003) and elevated level of *Prevotella* (*P* = 0.038) and *Roseburia* (*P* = 0.005) at the genus level.

**Conclusions:**

The present results evidenced altered microbiome composition in schizophrenia patients with central obesity and further suggested the role of the gender factor in the process of gut dysbiosis.

## Introduction

Schizophrenia is a severe psychiatric disorder associated with a wide range of symptoms including hallucinations, delusions, negative symptoms and cognitive dysfunctions (1). Morbidity and mortality are higher in individuals with schizophrenia than in the general population. Obesity and obesity-related conditions are common health problems in patients with schizophrenia (2). Higher incidences of cardiovascular diseases related to obesity and metabolic dysfunction account for premature deaths of schizophrenia patients (3), resulting in an average of 14.5 years of potential life lost compared with the normal population (4). The development of obesity and metabolic problem in schizophrenia patients was associated with multiple factors including less physical activity (5), smoking and poor diet (6). Moreover, antipsychotic medications were reported to contribute to the high prevalence of obesity in those with schizophrenia (2). In addition to physical and medical factors, gender difference was reported to be associated with antipsychotic-related metabolic dysfunction. McEvoy et al. found higher prevalence of metabolic dysfunction among females than males receiving antipsychotics (7). The gender-dependent discrepancies may be partly attributable to gender differences in pharmacokinetics (8). However, the mechanism behind gender effects on metabolic dysfunction in schizophrenia patients remained unclear.

Studies have investigated the relationship between gut microbiota and obesity. The ratio of Firmicutes to Bacteroidetes was higher in obese mice than in lean mice (9). In obese human subjects, similar microbiota patterns were also observed (10). Evidence has also accumulated supporting a role for gut microbiota in metabolic diseases. Larsen et al. found significant differences in composition of microbiota between adults with type 2 diabetes mellitus and non-diabetic adults. The proportions of phylum Firmicutes and class Clostridia were reduced in the diabetic compared with the non-diabetic group (11). Le Chatelier et al. reported that individuals with low bacterial richness were characterized by more marked overall adiposity (12).

Although antipsychotic-related metabolic dysfunction has aroused clinical concern, the relationship between antipsychotic-related obesity and gut microbiota has been investigated in only a few clinical studies. Bahr et al. compared between 18 male adolescents chronically taking Risperidone and antipsychotic-naïve same-sex controls and found decreased Bacteroidetes/Firmicutes ratio and significant BMI gain in subjects on long-term treatment with Risperidone (13). Using a cross-sectional study design, Flowers et al. investigated the microbial communities and alpha diversity in 117 adults [49 atypical antipsychotic (AAP)-treated, 68 non-AAP-treated] with bipolar disorder. Decreased Simpson diversity was found in AAP-treated females compared with non-AAP-treated females. Furthermore, *Akkermansia* was significantly decreased in non-obese patients treated with AAPs (14). Yuan et al. followed up 41 first-episode schizophrenia patients for 24 weeks to assess the influence of Risperidone treatment on metabolic parameters relative to microbiota composition. They observed positive correlation between changes in fecal *Bifidobacterium* spp. and changes in weight over 24 weeks of Risperidone therapy (15). Flowers et al. investigated gut microbiota of 37 adults diagnosed with bipolar disorder or schizophrenia and treated with an AAP or mood stabilizer. AAP-treated females exhibited reduced gut microbial diversity compared with non-AAP-treated females (16).

Clinical research to date illustrated changes in gut microbiota in patients treated with AAP toward that of an obesogenic profile (13–15). Moreover, gender-dependent microbial alteration has been observed in several studies (13, 14, 16). Nevertheless, most of these studies were performed in an outpatient setting and dietary differences in the study group may be a concern. As mentioned in previous studies, diet has significant effects on gut microbiota diversity (17). This study aimed to investigate the differences in gut microbiota composition between schizophrenia patients with normal body weight and central obesity in an inpatient setting with less variance in patients' diet intake. We hypothesized that gut microbiota composition would differ between schizophrenia patients with normal weight and central obesity.

## Materials and Methods

### Participants

Forty schizophrenia patients, including 20 patients with central obesity and 20 patients with normal weight were recruited from the inpatient units of the Tsaotun Psychiatric Center, Ministry of Health and Welfare, Nantou, Taiwan. Ethical approval was obtained from the Institutional Review Board of Tsaotun Psychiatric Center (IRB No. 106035). All patients provided written informed consent after complete description of the study by research assistants. Patients were enrolled into this study if they (1) were aged from 20 to 65 years, (2) fulfilled the diagnosis of schizophrenia according to the criteria of the Diagnostic and Statistical Manual of Mental Disorders-Fifth Edition (DSM-5) (18), and (3) remained symptomatic but without clinically significant fluctuation and their antipsychotic doses had not been changed for at least 3 months prior to this study. Subjects with waist circumstance ≥ 90 cm for men and ≥ 80 cm for women are classified as central obese (CO) while those with body mass index (BMI) of 18.5–24 and waist circumstance <90 cm for men and <80 cm for women are classified as normal weight (NW). Exclusion criteria included DSM-5 diagnosis of intellectual disability, dementia, substance/alcohol abuse or dependence, uncontrolled gastrointestinal disorders, antimicrobial exposure or probiotics use within 3 months, presence of medical conditions that would significantly affect weight changes and inability to follow protocol.

### Sociodemographic, Clinical and Biochemical Assessments

Sociodemographic features (i.e., gender, age) and antipsychotic medications were ascertained through clinical interview and review of medical records. Daily dosages of antipsychotic medication were converted to chlorpromazine equivalent (19). Clinical assessments were conducted using the Positive and Negative Syndrome Scale (PANSS) (20). The ratings were performed by a research psychiatrist experienced with rating scales. Waist circumference was measured with a measuring tape. Systolic and diastolic blood pressure (SBP and DBP) in resting state were recorded. BMI, waist hip ratio (WHR), percentage of body fat (PBF) were determined using the non-invasive body composition analyzer (InBody 230, Seoul, Korea). Biochemical parameters levels in serum including glucose, glycated hemoglobin, total cholesterol (TG), high density lipoprotein-cholesterol (HDL-C), low density lipoprotein-cholesterol (LDL), and triglyceride were also measured.

### Stool Collection and Sequencing

All participants provided a stool sample that was immediately frozen after collection at hospital and delivered in ice bags within 24 h *via* commercial transport to the laboratory (Feng Chi Biotechnology, Taipei, Taiwan). DNA was extracted from fecal samples using a QIAamp PowerFecal DNA Isolation Kit (Qiagen, Hilden, Germany). The isolated DNA aliquot was stored at −80 °C until sequencing.

The hypervariable V3-V4 regions of bacterial 16S rRNA genes were amplified for library construction. Sequencing of the amplicon DNA samples was performed at Feng Chi Biotechnology (Taipei, Taiwan) using the Illumina Miseq platform. Taxonomic classification of operational taxonomic units (OTUs) at the phylum to genus levels were conducted according to Greengenes database using QIIME1 software.

### Bioinformatics and Statistical Analyses

Demographic and clinical characteristics were compared using Student's *t*-tests for the continuous variables and chi-square tests for the discrete variables. The alpha-diversity, observed OTUs, Shannon index, Simpson index and inverse Simpson's Diversity, estimate of two groups were compared using independent *t*-tests. The beta-diversity comparison was performed using the principle coordinates analysis (PCoA) of Bray-Curtis distances. The between-groups inertia percentage was examined using the Monte-Carlo test. The Wilcoxon test was performed to identify the significance of taxonomy between the two groups.

## Results

### Patients' Characteristics

General characteristics of the subjects are presented in Table 1. As can be seen, there was no significance in age, chlorpromazine equivalent and PANSS total score between the CO and NW groups. As expected, CO subjects have larger BMI (*P* < 0.001) and waist circumstance (*P* < 0.001) as well as higher WHR, PBF, SBP, HDL-C, and TG. Gender differences in clinical characteristics of subjects were also observed. Male CO subjects have significantly higher WHR (*P* < 0.001), SBP (*P* < 0.05) and DBP (*P* < 0.01) than male NW subjects. However, the differences in WHR, SBP and DBP between female CO and NW subjects were not significant.

**Table 1:** Demographic and clinical characteristics of studied patients.

	**Male**				***p*-value**	**Female**				***p*-value**	**Total**					**Central** **obesity** ***N*** **=** **10**		**Normal** **weight** ***N*** **=** **10**			**Central** **obesity** ***N*** **=** **10**		**Normal** **weight** ***N*** **=** **10**			**Central** **obesity** ***N*** **=** **20**		**Normal** **weight** ***N*** **=** **20**		
	**M**	**SD**	**M**	**SD**		**M**	**SD**	**M**	**SD**		**M**	**SD**	**M**	**SD**	
Age (years)	50.1	7.5	46.8	11.9	0.23	52.2	7.5	50.6	6.0	0.30	51.2	7.4	48.7	9.4	0.18
CPZ equivalent (mg/d)	508.8	143.2	575.5	138.7	0.15	472.8	245.1	578.8	302.6	0.31	490.8	245.1	577.1	229.1	0.26
PANSS total score	86.5	8.7	86.1	6.3	0.45	86.9	6.7	87.1	5.7	0.47	86.7	6.7	86.6	5.9	0.48
BMI	26.7	2.2	19.9	1.7	<0.001	26.2	2.0	20.0	1.9	<0.001	26.4	2.0	20.0	1.8	<0.001
Waist circumference (cm)	96.9	4.2	78.7	5.8	<0.001	96.1	7.3	74.7	6.8	<0.001	96.5	7.3	76.7	6.5	<0.001
WHR	0.89	0.05	0.81	0.03	<0.001	0.89	0.05	0.84	0.08	0.058	0.89	0.05	0.83	0.06	<0.001
PBF (%)	26.0	6.0	15.5	6.1	<0.001	34.2	3.6	24.5	8.4	<0.01	30.0	6.4	20.0	8.5	<0.001
SBP (mmHg)	119.2	13.4	106.1	10.9	<0.05	111.6	11.2	104.9	7.0	0.06	115.4	11.2	105.5	8.9	<0.01
DBP (mmHg)	83.5	9.3	72.6	8.9	<0.01	68.8	9.6	69.5	5.9	0.42	76.2	9.7	71.0	7.6	0.05
AC sugar (mg/dl)	96.5	14.4	97.2	15.1	0.45	99.6	12.6	92.7	7.5	0.07	98.1	12.6	95.0	11.8	0.22
HbA1c (%)	5.4	0.6	5.5	0.3	0.38	5.6	0.5	5.37	0.3	0.11	5.5	0.5	5.4	0.3	0.29
Total cholesterol	159.2	28.0	161.2	28.8	0.44	182.3	40.0	192.1	40.8	0.30	170.8	40.0	176.7	37.8	0.31
HDL-C (mg/dl)	35.3	4.9	46.3	8.6	<0.01	42.0	9.4	54.4	18.0	<0.05	38.6	9.4	50.4	14.4	<0.01
LDL-C (mg/dl)	98.5	26.6	92.7	23.9	0.31	114.5	37.2	108.2	32.0	0.35	106.5	37.2	1,005	28.7	0.27
Triglyceride (mg/dl)	146.9	56.5	77.2	17.9	<0.001	142.7	49.1	98.4	42.6	<0.05	144.8	49.1	87.8	33.6	<0.001

*CPZ, Chlorpromazine; PANSS, Positive and Negative Syndrome Scale; BMI, body mass index; WHR, waist hip ratio; PBF, percentage of body fat; SBP, systolic blood pressure; DBP, diastolic blood pressure; AC, ante cibum; HDL-C, high density lipoprotein-cholesterol; LDL-C, low density lipoprotein-cholesterol; HbA1c, glycated hemoglobin*.

### Bacteria Community Structure

**Figure 1:**
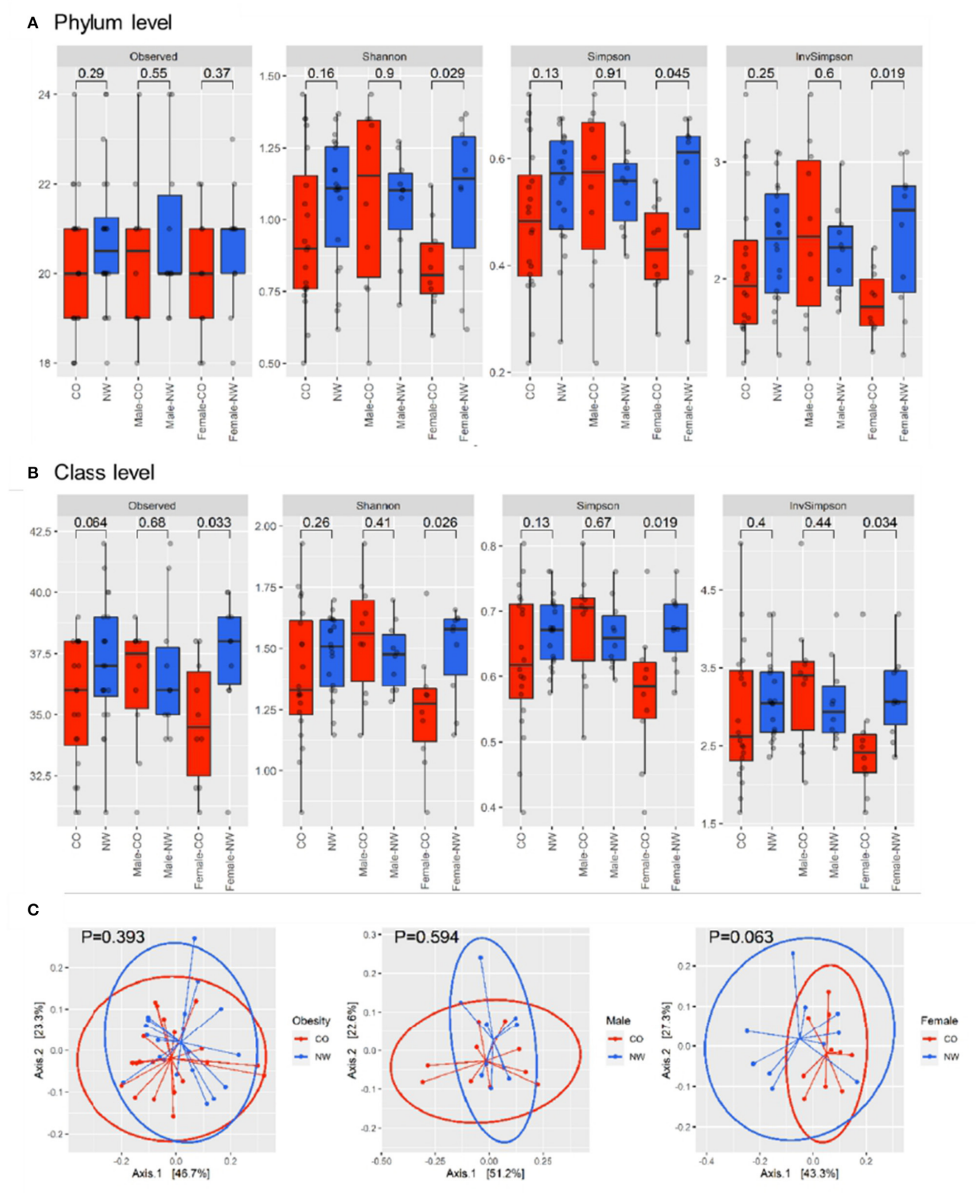
Alpha and beta diversity were compared between subjects with central obesity (CO) and normal weight (NW). Bacterial community richness was defined by the observed number of operational taxonomic units (OTUs) and alpha diversity was calculated using the Shannon index, Simpson index, and inverse Simpson index for both phylum **(A)** and class levels **(B)**. The analysis of beta diversity was calculated using PCoA of Bray-Curtis distances **(C)**.

Comparison between the CO and NW groups showed no significant difference in alpha diversity, number of observed OTUs, Shannon index, Simpson index, and inverse-Simpson index at both phylum (Figure 1A) and class levels (Figure 1B). The analysis of beta diversity calculated using PCoA of Bray-Curtis distances revealed no distinction in composition of gut microbiota between CO and NW subjects (Figure 1C). As for gender-based differences of gut microbiota, female NW subjects had significantly higher richness of gut microbiota at the class level (*P* = 0.033) (measured by the number of observed OTUs) and higher alpha diversity at both phylum and class levels (measured by the Shannon, Simpson, and Inverse-Simpson indexes) compared with female CO subjects (Figures 1A,B). As for beta diversity, the two groups showed distinct gut microbiota composition at the phylum level (*P* = 0.063) (Figure 1C). In contrast, there was no difference in measures of alpha diversity and beta diversity between male CO and NW subjects.

### Taxonomic Profiles Comparison

**Figure 2:**
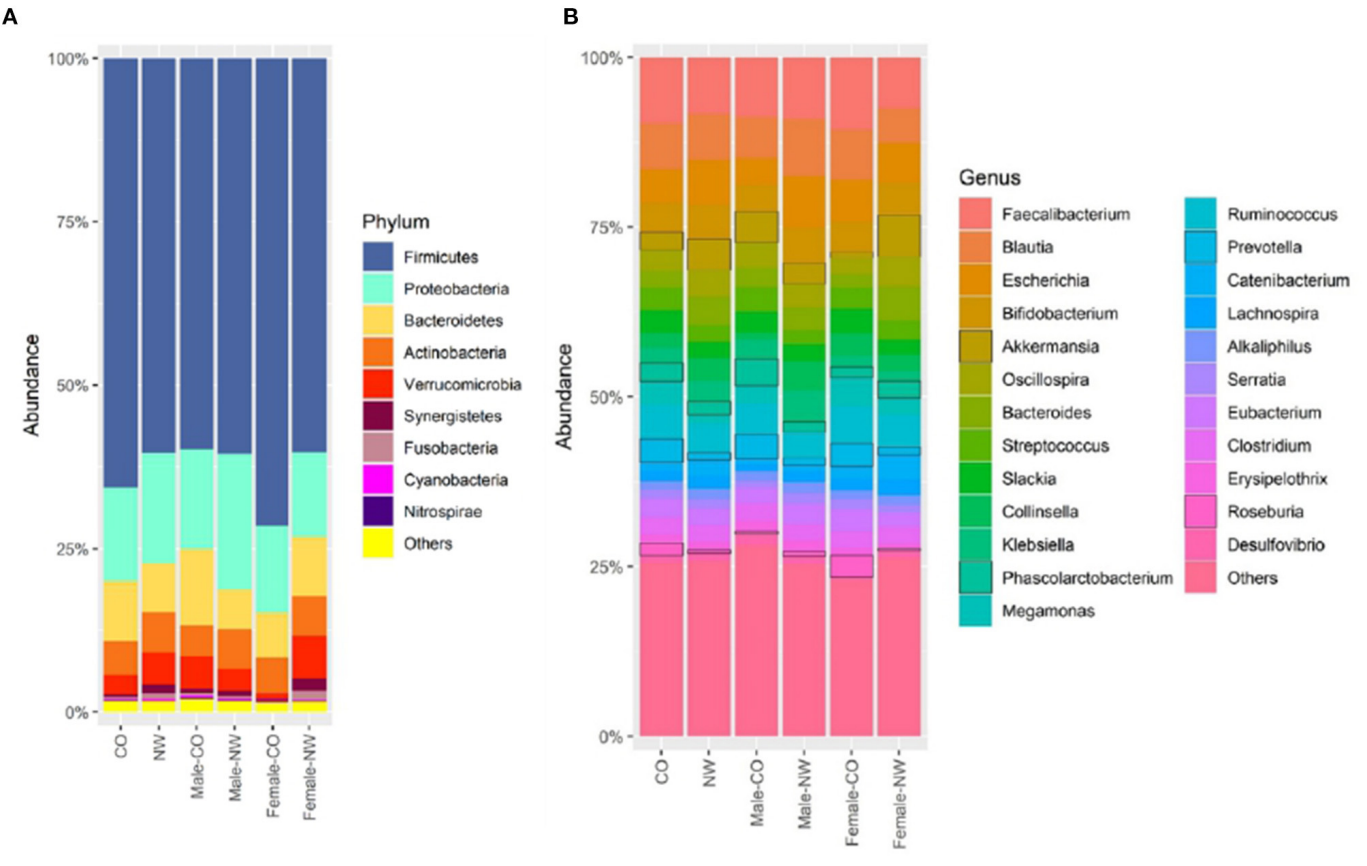
Relative abundance of operational taxonomic units (OTUs) of the gut microbiota of central obese (CO) and normal weight (NW) subjects at **(A)** phylum and **(B)** genus levels.

**Figure 3:**
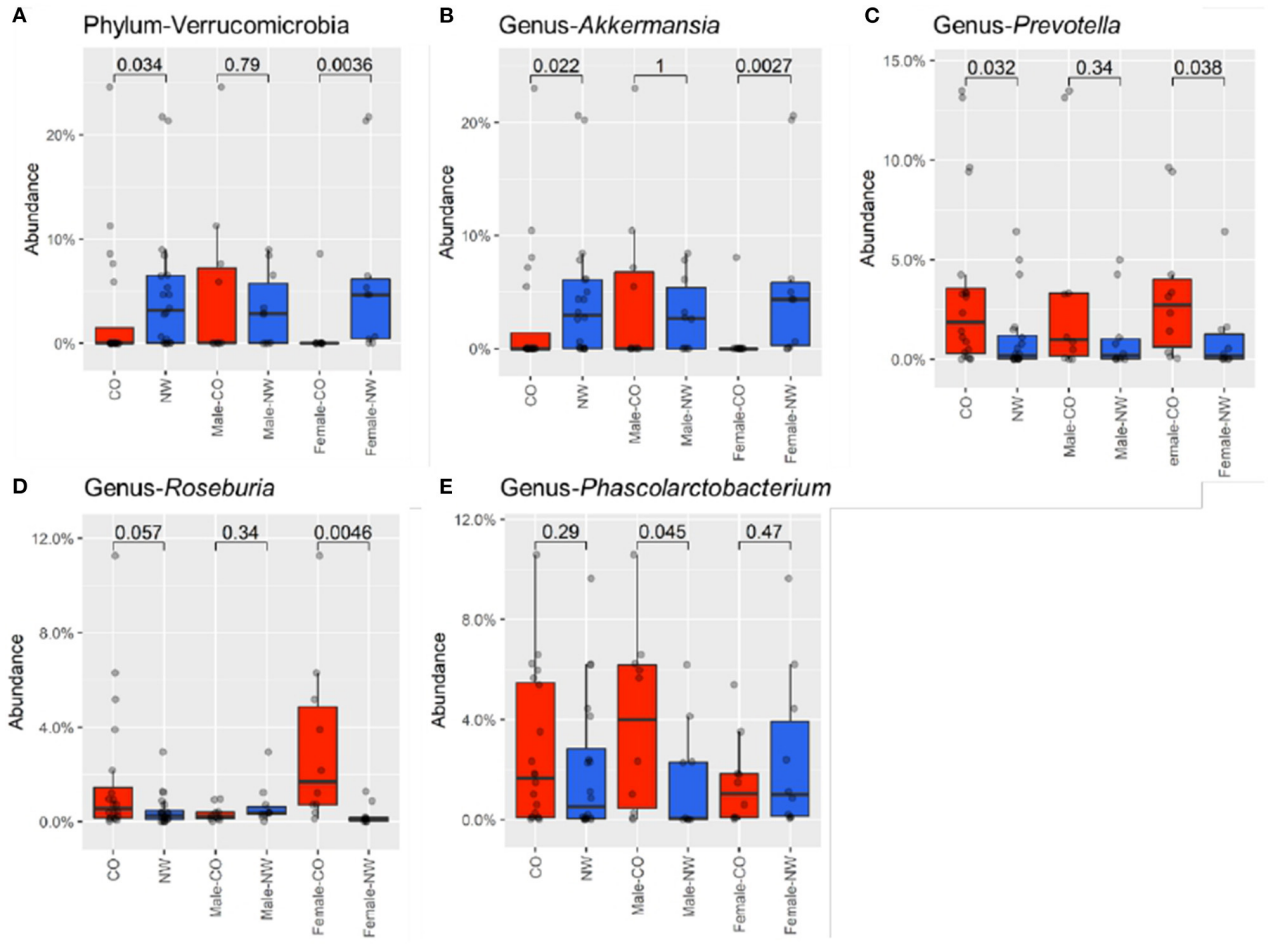
Relative abundances were compared between male and female subjects with central obesity (CO) and normal weight (NW). The relative abundance of Verrucomicrobia at phylum **(A)**, genus of **(B)** *Akkermansia*, **(C)** *Prevotella*, **(D)** *Roseburia*, and **(E)** *Phascolarctobacterium* were compared. Female CO subjects had significantly lower abundance of Verrucomicrobia at the phylum level. At the genus level, relative abundance of *Akkermansia* was decreased in female CO subjects compared with female NW subjects, while *Prevotella* and *Roseburia* were increased in female CO subjects. *Phascolarctobacterium* was enriched in male CO subjects compared with male NW subjects.

Taxonomic analysis was performed to assess the composition of gut microbiota at different levels in samples of CO and NW subjects. This study found less Bacteroidetes than Firmicutes in the schizophrenia subjects (Figure 2A). Figure 2B shows the relative abundance of gut microbiota in CO and NW subjects at the genus level. At the phylum level, CO subjects showed significantly decreased abundance of Verrucomicrobia compared with NW subjects (*P* = 0.034) (Figure 3A). At the genus level, CO subjects of both genders showed significantly decreased abundance of *Akkermansia* (*P* = 0.022) (Figure 3B) and increased abundance of *Prevotella* (*P* = 0.032) (Figure 3C) compared with their NW counterparts.

Differences in taxonomic profile was observed mainly among female subjects. Female CO subjects had significantly lower abundance of Verrucomicrobia (*P* = 0.004) (Figure 3A) at the phylum level. At the genus level, three differentially abundant genera were observed. Relative abundance of *Akkermansia* was reduced in CO subjects compared with NW subjects (*P* = 0.003) (Figure 3B), while *Prevotella* (*P* = 0.038) (Figure 3C) and *Roseburia* (*P* = 0.005) (Figure 3D) were elevated in CO subjects.

Comparison among male subjects showed no significant difference in taxonomic profile at the phylum level. Only the *Phascolarctobacterium* genus was found to be enriched in male CO subjects compared with male NW subjects (*P* = 0.045) (Figure 3E).

## Discussion

### Relationships With Previous Studies

This study aimed to investigate the differences in microbiota composition between schizophrenia patients with normal body weight and central obesity. We found lower bacterial richness and alpha diversity in female CO subjects than in female NW subjects. In a rat model, olanzapine was found to induce significant body weight gain and to reduce gut microbial diversity in female rats only (21). Bahr et al. observed an increase in body weight and microbiota diversity in male adolescents treated with Risperidone (13). Flowers et al. found that treatment using second-generation antipsychotics (SGA) was associated with weight gain and reduction in microbial species richness in females (14, 16). The present findings also suggest the role of the gender factor in antipsychotic-related gut dysbiosis.

The findings of bacterial taxonomic composition were diverse across studies. In this study, only female subjects had different bacterial taxonomic composition. Flowers et al. found significantly reduced *Akkermansia* in non-obese female bipolar patients treated with AAPs (14). However, the current results showed decreased *Akkermansia* in CO subjects compared with NW subjects. *Akkermansia* is a genus in the phylum Verrucomicrobia containing a single known species, namely *Akkermansia muciniphilia* which was found in the human gut (22). *A. muciniphilia* is an intestinal mucin degrader and was found to regulate metabolic functions and considered a promising next-generation probiotic (23, 24). Decreased abundance of *Akkermansia* in CO subjects was notable in this study. Moreover, *Prevotella* and *Roseburia* in female CO subjects and *Phascolartobacterium* in male CO subjects were more abundant compared with NW subjects. Bahr et al. found less abundant *Prevotella* in chronic Risperidone-treated adolescents with significant BMI gain than those without BMI gain (13). Li et al. noted lower relative abundance of *Roseburia* in schizophrenia patients than in normal control subjects (25). Shen et al. observed increased relative abundance of *Phascolarctobacterium* in schizophrenia subjects (26). Further investigations are necessary to elucidate the role of the above-mentioned taxa in schizophrenia patients with central obesity.

The lack of significant difference in bacteria diversity between the CO and NW groups may partly be attributed to their relatively minor BMI difference. Some studies reported sex hormone as a factor determining gut microbiota composition. Reduced abundance of *Akkermansia* was reported to be correlated with higher levels of follicule-stimulating hormone (FSH) and luteinizing hormone (LH). FSH level was reported to be positively correlated with abundance of *Roseburia* and *Prevotella* (27). Another study reported that post-menopausal women had lower relative abundance of *Prevotella* than pre-menopausal women (28). Different abundance of the abovementioned three genera were observed in this study. Although the levels of sex hormones were not analyzed, considering the subjects' age (mean, 51.4 years), the alteration of gut microbiota, especially in female subjects, might be associated with hormonal change. Future investigations should further assess the relationship of sex hormone with gut microbiome in schizophrenia patients.

### Limitations and Highlights

This study has several limitations. The sample size was small; hence, the findings can only be considered preliminary and cannot be further extrapolated. The cross-sectional design hinders drawing valid conclusions on the role of gut microbiota in CO schizophrenic subjects. Although CO subjects in this study had significantly higher BMI than NW subjects (26.4 vs. 20.0, *P* < 0.001), the average BMI of CO subjects was 26.4, which was overweight in terms of BMI classification. We did not recruit normal control subjects in current study. In future work, it may be prudent to include microbiota composition of normal control subjects to better outline the microbiota changes in schizophrenia patients.

Despite the limitations mentioned above, this study investigated differences in gut microbiota composition of schizophrenia patients that are relatively homogeneous in their dietary variance. Some gender-related differences in gut microbiota were observed. Future longitudinal and large-scale studies are required to further elucidate the interplay between metabolic problems and microbiota in schizophrenia patients. The current results highlight the importance of considering gender as a variable factor when examining interactions among microbiota, central obesity and metabolic dysfunction in this population.

## Conclusion

The study results evidenced altered microbiome composition in schizophrenia patients with central obesity and further suggested the role of the gender factor in the process of gut dysbiosis.

## Data Availability Statement

The datasets presented in this study can be found in online repositories. The names of the repository/repositories and accession number(s) can be found at: https://www.ncbi.nlm.nih.gov, accession ID: PRJNA790710.

## Ethics Statement

Ethical approval was obtained from the Institutional Review Board of Tsaotun Psychiatric Center (IRB No. 106035). All patients provided written informed consent after complete description of the study.

## Author Contributions

Y-LT and Y-WL conceived and designed the study, and wrote the manuscript. C-YL and T-HL coordinated the progress of the study, and revised the manuscript. P-NW assisted for participants' referral and recruitment. All authors contributed to the article and approved the submitted version.

## Funding

The study was funded by Tsaotun Psychiatric Center, MOHW (Grant number: 110004) and Hospital and Social Welfare Organizations Administration Commission, MOHW (Grant number: 10744). The funders were not involved in design and conduct of the study; collection, management, analysis, and interpretation of the data; preparation, review, or approval of the manuscript; and decision to submit the manuscript for publication.

## Conflict of Interest

The authors declare that the research was conducted in the absence of any commercial or financial relationships that could be construed as a potential conflict of interest.

## Publisher's Note

All claims expressed in this article are solely those of the authors and do not necessarily represent those of their affiliated organizations, or those of the publisher, the editors and the reviewers. Any product that may be evaluated in this article, or claim that may be made by its manufacturer, is not guaranteed or endorsed by the publisher.
