# Correction: Gender differences in gut microbiome composition between schizophrenia patients with normal body weight and central obesity

**DOI:** 10.3389/fpsyt.2026.1805447

**Published:** 2026-02-25

**Authors:** Yun-Lin Tsai, Yen-Wenn Liu, Peng-Nien Wang, Chun-Yuan Lin, Tsuo-Hung Lan

**Affiliations:** 1 Tsaotun Psychiatric Center, Ministry of Health and Welfare, Nantou, Taiwan; 2 Institute of Biochemistry and Molecular Biology, National Yang Ming Chiao Tung University, Taipei, Taiwan; 3 National Changhua University of Education, Changhua, Taiwan

**Keywords:** schizophrenia, microbiome, central obesity, gender difference, gut dysbiosis

Affiliation 3 “National Changhua University of Education, Changhua, Taiwan” was omitted from the paper. This affiliation has now been added for author “Chun-Yuan Lin”.

National Changhua University of Education is a non-profit academic institution. The addition of the new affiliation does not affect the **Conflict of interest** statement; it remains as previously declared. The authors declare that the research was conducted in the absence of any commercial or financial relationships that could be construed as a potential conflict of interest.

The original article has been updated.

